# A Further Report on the Treatment of Pellagra

**Published:** 1911-07

**Authors:** Geo. M. Niles

**Affiliations:** Atlanta, Ga.; Professor of Physiology, Southern College of Pharmacy; Associate Professor of Physiology, Atlanta School of Medicine; Gastro-Enterologist to the Tabernacle Infirmary


### A FURTHER REPORT ON THE TREATMENT OF PELLAGRA.

BY GEO. M. NILES, M.D., ATLANTA, GA.

*Professor of Physiology, Southern College of Pharmacy; Associate Professor of Physiology, Atlanta School of Medicine; Gastro-Enterologist to the Tabernacle Infirmary.*

In July, 1910, I wrote an article entitled "Some Remarks on the Treatment of Pellagra," which was published in the New York Medical Record, Sept. 10th. In this article I sounded rather an optimistic note, and, notwithstanding the jeremiads of a few discouraged practitioners, smarting under some recent lost cases, I still feel that we have much more reason for encouragement than otherwise.

The etiology is not yet settled. The zeists and anti-zeists are still waging their learned warfare; the simulium reptans is under grave suspicion by some; while a new Richmond has recently entered the lists, and shied his castor in the ring with the theory that pellagra is caused by semi-drying edible oils.

Being a man of peace, I decline to enter this wordy battle, though, giving the maize theory the benefit of the doubt, I omit corn products from the dietary of my pellagrins.

My present treatment has been evolved from the composite ideas of others, coupled with my own, and at present these methods are serving me so well, that I would hesitate to drop them for any other line of treatment, unless supported by very strong clinical proof.

Two years ago I used atoxyl hypodermically and Fowler's solution internally as the basis of treatment. I then dropped the atoxyl, substituting the iron arsenite solution in sixteen minim ampules. A little later I began giving internally a combination of saturated solution of k. i. and Fowler's solution in the proportion of 5 to 3, as suggested by Dr. R. T. Dorsey. In the early part of this year I included in my treatment the cacodylate of soda, in 1 c. c. ampules, each containing three-fourths of a grain of the drug.

At present I use the iron arsenite solution and the cacodylate of soda on alternate days, injecting them under careful aseptic precautions, and giving one daily for one or two weeks. After that, I give an ampule every second day, but still alternate them. After the symptoms seem to be controlled, I continue giving one injection weekly (still alternating them) for three or four months.

Internally, I use the combination of k. i. and Fowler's solution, as indicated previously, beginning with five drops in water daily after meals, and increasing one drop daily until symptoms of arsenical saturation are manifested. This generally comes on when from twenty to thirty drops of the combination are being taken. When there is puffiness under the eyes on arising, I stop the drops for two days, beginning again at the minimum dose of five drops, and increasing one drop daily as before. This procedure I continue until the eruption and sore mouth are abated; and then keep it up in eight drop doses for several months. Should there arise a gastric or intestinal intolerance, a condition occasionally in evidence, it may be necessary to reduce the proportion of Fowler's solution to one or two in eight parts, instead of three as originally suggested.

For the frequent diarrhea I have obtained satisfaction from bismuth-betanaphtol and resorcin, given with milk of bismuth as a vehicle. This failing, I give tannigen, protan, or heavy doses of bismuth subgallate, or, as a last resort, powdered opium.

For the occasional constipation, either castor oil or enemas will serve, drastic cathartics being inadmissible.

For the sore mouth, a solution of thymol, one grain to the ounce of water, a little alcohol being used for a solvent, will generally prove sufficient. Also a 25 per cent. solution of boroglycerin is helpful. For the aphthous spots in the mouth or lips or on the tongue, touching with silver nitrate (twenty grains to the ounce of water) is in most instances efficacious.

Where there is a paucity or absence of free hydrochloric acid in the stomach contents, a lavage every alternate day with a 1-100 solution of boracic acid, sodium salicylate, thymol, or ichthyol may be employed to advantage. When these medicated douches are used, however, it is well to start with a mild saline solution, then the medicated fluid, then conclude with plain water.

Special treatment of the mental symptoms, appearing as they do in such Protean shapes, will not be discussed in this paper, as it would lead into a branch of therapeutics in which I would defer to the alienists.

The simple erythematous rashes on up to sloughing conditions of the hands or feet may be alleviated or cured by the bland ointments, such as oxide of zinc ointment or a 5 per cent. ointment of boric acid. Avoidance of the sun's direct rays, or even very bright light is advisable during the continuance of the erythema. For the intense burning of the hands and feet, so often and bitterly complained of, either compresses saturated with a mild solution of bichloride of mercury, ice cold, and applied at frequent intervals, or baths in hot mustard water will afford marked relief.

Dietetic regulations depend altogether on the state of the gastro-intestinal tract, or as to the presence of constipation or diarrhea, and need not vary materially from the same regime instituted for the same manifestations of digestive disquietude set up by other disease. Speaking generally, a liberal diet is indicated, the flesh proteins being especially well borne. I also venture the opinion, which opinion is not yet widely accepted, that the diarrhea appearing early in the course of pellagra is of central origin, and, to an extent compensatory. It would naturally follow, therefore, that a diarrhoea of this sort need not entail a too limited dietary. Later on, when the diarrhoea has become inflammatory, the physician may to advantage eliminate articles of food containing much cellulose, or yielding after digestion a residue of irritating particles. In any event, so far as is consistent with local conditions, the patient should be nourished to the limit of assimilation.

Other symptoms may be treated on their merits as they arise.

In consulting my case records I find one pellagra patient who has been well over two years, and nine who have shown no symptoms for over one year. I also note four deaths, one being a relapse about three weeks after I had dismissed him, following a drunken debauch.

In addition, I have quite a number who have seemed well for a shorter length of time, or seem to be improving, but do not think it wise to include them as cured until a year or more has elapsed.

From my point of view, which is, of course, open to human fallability, I feel that this line of treatment holds much of promise, and, for the present, at least, I shall continue to work along the therapeutic principles, as indicated in this brief report.

920-21 Candler Building.

---

### SURGICAL SUGGESTIONS.

Septic endocarditis may result from a localized osteomyelitis that has gone on to spontaneous cure.

Don't abandon a case of sarcoma as incurable without a thorough trial of Coley's fluid. The results are sometimes remarkable.

Broad sessile warts are best removed by the application, under water, of the Oudin high frequency current by contact with the tip of an insulated copper conducting cord.

